# Investigation of an *Escherichia coli *O145 outbreak in a child day-care centre - extensive sampling and characterization of *eae*- and *stx*_1_-positive *E. coli *yields epidemiological and socioeconomic insight

**DOI:** 10.1186/1471-2334-11-238

**Published:** 2011-09-08

**Authors:** Erik Wahl, Line Vold, Bjørn A Lindstedt, Torkjel Bruheim, Jan E Afset

**Affiliations:** 1 District Office Trondheim and Orkdal, Norwegian Food Safety Authority, PO Box 383, N-2381 Brumunddal, Norway; 2 Department of Infectious Disease Control, Norwegian Institute of Public Health, PO Box 4404, Nydalen, N-0403 Oslo, Norway; 3 National Veterinary Institute, PO Box 5695 Sluppen, N-7485 Trondheim, Norway; 4 Department of Medical Microbiology, Laboratory centre, St Olavs University Hospital, N-7006 Trondheim, Norway; 5 Department of Medical Microbiology, St Olavs University Hospital, N-7006 Trondheim, Norway

## Abstract

**Background:**

On October 29^th ^2009 the health authorities in the city of Trondheim, Norway were alerted about a case of Shiga toxin-positive *E. coli *(STEC) O145 in a child with bloody diarrhoea attending a day-care centre. Symptomatic children in this day-care centre were sampled, thereby identifying three more cases. This initiated an outbreak investigation.

**Methods:**

A case was defined as a child attending the day-care centre, in whom *eae- *and *stx*_1_- but not *stx*_2_-positive *E. coli *O145:H28 was diagnosed from a faecal sample, with multilocus variable number of tandem repeat analysis (MLVA) profile identical to the index isolate. All 61 children, a staff of 14 in the day-care centre, and 74 close contacts submitted faecal samples. Staff and parents were interviewed about cases' exposure to foods and animals. Faecal samples from 31 ewes from a sheep herd to which the children were exposed were analyzed for *E. coli *O145.

**Results:**

Sixteen cases were identified, from which nine presented diarrhoea but not haemolytic uremic syndrome (HUS). The attack rate was 0.26, and varied between age groups (0.13-0.40) and between the three day-care centre departments (0.20-0.50), and was significantly higher amongst the youngest children. Median duration of shedding was 20 days (0-71 days). Children were excluded from the day-care centre during shedding, requiring parents to take compassionate leave, estimated to be a minimum total of 406 days for all cases. Atypical enteropathogenic *E. coli *(aEPEC) were detected among 14 children other than cases. These isolates were genotypically different from the outbreak strain. Children in the day-care centre were exposed to faecal pollution from a sheep herd, but *E. coli *O145 was not detected in the sheep.

**Conclusions:**

We report an outbreak of *stx*_1_- and *eae-*positive STEC O145:H28 infection with mild symptoms among children in a day-care centre. Extensive sampling showed occurrence of the outbreak strain as well as other STEC and aEPEC strains in the outbreak population. MLVA-typing of the STEC-isolates strongly indicates a common source of infection. The study describes epidemiological aspects and socioeconomic consequences of a non-O157 STEC outbreak, which are less commonly reported than O157 outbreaks.

## Background

*Escherichia coli *is part of the normal microflora in the intestinal tract of humans and warm-blooded animals [1]. Shiga toxin-producing *E. coli *(STEC) are associated with hemorrhagic colitis and haemolytic uremic syndrome (HUS), which can lead to renal failure and death. This occurs more often among children younger than 10 years compared to adults [2,3]. An essential part in STEC-pathogenesis is the ability to secrete Shiga toxins 1 and/or 2, encoded by the genes *stx*_1 _and *stx*_2_. Hemorrhagic colitis, HUS and other serious symptoms or adverse effects are considerably more frequent among cases infected by *stx*_2_-positive, compared to *stx*_1_-positive STEC [1,4]. *E. coli *strains are grouped into serotypes based on O (somatic) and H (flagellar) antigens. More than 600 serotypes of STEC have been identified. STEC O157:H7 caused several large outbreaks in North America, Europe and Japan in the 1980s and 1990s. More recently, several non-O157 STEC serotypes, such as O26, O103, O111 and O145 have also been associated with diarrhoea, haemorrhagic colitis and HUS [5]. Routine diagnosis and surveillance of STEC-infections was originally developed for serotype O157. However, non-O157 *E. coli *infections are considered in certain geographic regions to be at least equally important, but may be underdiagnosed [4]. The *stx *genes are located in the bacterial genome on a lambdoid prophage, but may be excised from the chromosome if the prophage is induced and enters a lytic phase [6,7]. Loss of *stx *genes has been demonstrated during subculture in the laboratory [8], and has been made probable *in vivo *in epidemiological studies [9,10]. Such loss of *stx *genes from the bacterium may make it difficult to distinguish between STEC and related atypical enteropathogenic *E. coli *(aEPEC). aEPEC are characterized similarly to most STEC by the ability to cause attaching and effacing lesions, for which the *eae *gene is essential, but lack *stx*-genes and the EPEC adherence factor (EAF)-plasmid with the bundle-forming pilus *bfpA *gene characteristic of typical EPEC [11,12].

Human hosts are normally infected by STEC through a faecal-oral transmission route. Domestic ruminant intestine is considered to be the most important reservoir for human infections of both O157 and non-O157 STEC [13]. European studies report STEC prevalence in healthy cattle ranging from 10 to 50% and in sheep from 36 to 67% [14-16]. Preliminary data from an unpublished prevalence-study in Norway showed presence of *eae*-positive *E. coli *O145 in 21 of 45 (47%) sheep herds, representing approximately 2500 sampled individuals. None of these isolates were *stx*-positive (personal communication Bruheim T.). Contamination within the food-chain, faecally contaminated drinking water and direct or indirect animal contacts are considered to be dominant routes for human infections, the latter affecting mainly young children [1]. Extraordinarily low infection dose, as low as < 45 cells in one reported *E. coli *O157 outbreak [17], and persistence in natural environment and food items [18,19], contribute to a high spreading potential within the human population.

In Norway (total population: 4.9 million), an average of 25 domestically acquired STEC human cases have been notified annually during the last decade. Among reported Norwegian cases of *E. coli*-infections in 2009 (including both STEC and aEPEC), several separate local outbreaks were identified [20]. In addition to the outbreak described here, one similar outbreak was reported this year involving five children in a day-care centre infected with aEPEC O76, where contact with calves during a farm visit was the suspected source of infection. STEC has been identified, not only as the most important causal agent for HUS and bloody diarrhoea [21], but also as a causal agent for mild and moderate gastrointestinal disease in America and Europe [4,22,23].

According to Norwegian national guidelines, children attending day-care centres who are infected with STEC should be kept away from the day-care centre until they have presented three to five consecutive negative faecal samples [24]. Until recently, these guidelines have not taken into account the type of STEC that was found.

*eae*-positive- and *stx- *and *bfpA-*negative *E. coli *(aEPEC) is more prevalent compared to STEC in industrialized countries, and is frequently detected in both children with diarrhoea and in healthy children [11,25,26]. Gastrointestinal disease is common in the general population, but more prevalent among children up to five years compared to the general population [27,28]. However, the impacts of both STEC as well as aEPEC on endemic non-bloody gastrointestinal disease and outbreaks in industrialized countries are not clear.

Multilocus variable number of tandem repeat analysis (MLVA) has proven to be useful for characterizing the clonal structure of *E. coli *and other pathogens in both outbreak [29] and endemic [30] investigations.

On October 9^th ^2009, the municipal health officer in the city of Trondheim, Norway, was alerted by St Olavs University Hospital, of a three year old child with STEC infection and bloody diarrhoea, who fell ill on September 28^th ^(index case). This child attended a day-care centre, which was informed, and faecal samples were collected from all children in the day-care centre with any history of diarrhoea during the preceding weeks. Among these, three new cases of STEC infections were identified. On this basis, the Municipal Health Officer declared this a possible outbreak associated to the day-care centre, and initiated an investigation in cooperation with the Norwgian Institute of Public Health, St Olavs University Hospital Department of Medical Microbiology, and the Food Safety Authority. The objectives were to describe the outbreak, to identify the source and, if possible, advise on and implement relevant control measures. We describe here the outbreak investigation and the results of the extensive sampling which this cross-sectional study led to, focusing on descriptive epidemiological aspects, microbial diversity and possible sources of infection.

## Methods

### Case definition

A case was defined as a child attending the day-care centre in September and October 2009, in whom *eae *and *stx*_1_-positive *E. coli *O145:H28 with a MLVA profile identical to that of the index isolate (outbreak-profile) was diagnosed from a faecal sample.

### Investigations of day-care centre environment

Environmental investigation of the day-care centre included inspection of the premises and interviews with staff, aimed at identifying potential sources of STEC infection in the day-care centre. During the interviews we used standard questionnaires and asked questions related to general food handling and hygienic procedures, as well as recognized specific risk factors [1] including exposure to raw minced meat, cured meat sausage, unpasteurized dairy products, potentially faecally contaminated vegetables, and non-disinfected water as well as animal contacts. We asked about exposures during the period September 1^st ^to September 28^th^. The staff was asked to list all foods served to children during stay in the day-care centre in this period. Questions on animal contacts included both direct and indirect contacts on regular or accidental basis. Investigation also focused on risk factors and procedures associated to risk of person to person transmission within the day-care centre, such as hand washing, procedures for napkin change, toilet assistance and disinfection of toys, as well as child contact patterns.

### Case interviews

For each case, a parent was interviewed by telephone, using a simple questionnaire, covering demographic data, clinical symptoms and exposures to the same risk factors during the same period as the investigation in the day-care centre.

### Faecal sampling from humans and case finding

After diagnosis of the index case on the 9^th ^of October, children and staff in the day-care centre who had experienced an episode of diarrhoea during the last weeks, were asked on the 12^th ^of October to submit faecal samples. As a response to this request, 16 children submitted faecal samples. When STEC O145 was later identified in samples from three children from this group, all children and staff who had not yet submitted a faecal sample, were asked on the 19^th ^of October asked to submit faecal samples. This group included the remaining 45 children and all 14 members of staff of the day-care centre. Thus, by the third week of October, all 61 children and all 14 staff members in the day-care centre had submitted faecal samples. All close contacts to cases were also asked to submit faecal samples, including all cases' household members and all others who had stayed in cases' households for one day or more, or anyone who had changed diapers for cases. Altogether 74 close contacts submitted faecal samples.

Faecal samples were cultured on Sorbitol MacConkey agar and incubated 24 hours at 35°C. For PCR analysis a 2-3 cm streak of mixed growth was suspended in 4 ml physiological saline, diluted 1:100, and extracted on a Qiagen BioRobot M48 instrument using MagAttract DNA Mini 48 Kit (Qiagen, Hilden, Germany). PCR for the *eae *(single PCR), *stx*_1 _and *stx*_2 _(multiplex PCR) genes was done by an in-house real-time TaqMan assay containing forward and reverse primers (0.3 μM), probe (0.3 μM), 10 μl of PerfeCta Multiplex qPCR SuperMix, UNG with Accustart DNA polymerase (Quanta Biosciences, MD, USA), 5 μl of DNA template and dH_2_O in a total reaction mix of 20 μl, using oligonucleotide primers and probes as listed in Additional file 1.

Amplification was carried out with initial denaturation at 45°C (3 min) and 95°C (5 min), then 45 cycles (*eae*) or 40 cycles (*stx*_1_/*stx*_2_) with 10 sec at 95°C, 10 sec at 50°C (*eae*) or 58°C (*stx*_1 _and *stx*_2_) and 20 sec at 72°C, followed by cooling for 40 sec, on BioRad C1000 Thermal Cycler with CFX96 Real-Time PCR Detection System (BioRad, CA, USA) or Lightcycler 2.0 Real-Time PCR System (Roche Applied Science, Basel, Switzerland).

If PCR was positive in mixed culture from a stool sample, subcultures from twelve (*stx*_1_, *stx*_2_) or four (*eae) *single colonies from the primary agar plate were retested by PCR. s*tx- *and/or *eae*-positive isolates were biochemically confirmed as *E. coli *by Api 20E (BioMerieux, Marcy l'Etoile, France) and agglutinated with Anti-Coli O145 antiserum and other common STEC antisera (SIFIN, Germany). If a positive result from mixed culture was not confirmed in pure culture, the finding was confirmed by PCR analysis of a separate aliquot from the same mixed culture. Initial *stx*_1 _PCR-positive primary faecal cultures from which an *stx*_1_-positive *E. coli *could not be retrieved by retesting of single colonies were concentrated for *E. coli *O145 by immunomagnetic separation (AIMS) using Dynabeads™ and BeadRetriever™, as described by the manufacturers (Dynal Invitrogen, Oslo, Norway). The immunoconcentrate was plated onto MacConkey agar and/or blood agar and incubated 24 hours at 37°C for colony isolation, and thereafter single colonies were analysed for *eae *and *stx *by PCR. AIMS aimed at other serotypes than O145 was not performed.

All *eae-*, *stx*_1_- and/or *stx*_2_-positive *E. coli *isolates were submitted to the National Reference Laboratory for Enteropathogenic Bacteria at the Norwegian Institute of Public Health for confirmation, O:H serotyping, genotyping by MLVA as previously described [30], and confirmation of PCR results including analysis for the *bfpA *gene [31]. A dendrogram was constructed using Bionumerics v5.10 software (Applied-Maths, Sint-Martens-Latem, Belgium) using Categorical coefficients and the Ward algorithm [32].

Children in the day-care centre with positive findings of STEC in faecal samples were asked to submit follow-up samples once every week until the results of testing were negative, and thereafter every two days until five consecutive samples were negative, according to guidelines from the Norwegian Institute of Public Health [24]. Duration of faecal shedding was estimated as time interval between the first and last positive sample.

### Faecal sampling of sheep

Rectal faecal samples were collected on October 21st from 38 of a total of 60 ewes from the sheep herd to which the children from the day-care centre had been exposed. Basic data on sheep herd management were collected. Faecal samples were diluted 1:10 with buffered peptone water (BPW) and pre-enriched for 18-24 hours at 41.5°C. AIMS was used for concentration of *E. coli *O145 as described above. The immunoconcentrate was plated onto MacConkey agar and bovine blood agar for colony isolation and incubated for 24 hours at 35°C. Up to 10 colonies showing a typical coliform appearance were tested by slide agglutination with O145 antiserum (SIFIN, Germany), and up to five presumptive positive colonies were subcultivated onto blood agar plates. From the blood agar plates *E. coli *was confirmed by indole reaction and the isolates were tested for autoagglutination. AIMS aimed at other serotypes than O145 was not performed.

### Descriptive epidemiology

Demographic, clinical and microbiological data from all cases were compiled to investigate descriptive epidemiological parameters. To investigate association between age and attack rate, a Fisher's exact test analysis was conducted using aggregated age groups 1-3 years, and 4-5 years of age.

### Meteorological data

Data on daily precipitation in Trondheim city (Voll station) for the period 12^th ^to 25^th ^of September 2009 were obtained from http://eklima.met.no.

### Ethical considerations

Written consent for participation in the study were collected from parents of all cases presented in this report, and from the day-care centre, as advised by the Regional Medical Research Ethics Committee, Central Norway. The work presented in this report was carried out as part of an outbreak investigation. Testing of close contacts is routine in Norway in outbreak settings where the suspected agent is STEC. The Norwegian Institute of Public Health has general consent to conduct outbreak investigations. In case of suspicions of food or animals as source of infections, the Norwegian Food Safety Authority has general consent to contribute. A specific approval for the work done in this outbreak investigation was therefore not needed.

## Results

### Basic outbreak population data

The day-care centre had a total of 61 children aged one to five years, and a staff of 14, and was divided into three departments, termed departments 1-3, of which department 1 was reserved for children one to two years of age. Some of the children attended part time and were not present all weekdays.

### Microbial diversity in the tested population

*stx*_1_- and *eae-*positive *E. coli *O145:H28 in pure culture, in accordance with the case definition, was detected in 16 children. In addition, *stx *genes were detected in samples from three more children in the day-care centre. From one of these children an *eae*, *stx*_1_- and *stx*_2 _-STEC O? was detected in pure culture. From two children *eae *and *stx*_1_, and *eae *and *stx*_2_, respectively, were detected in primary mixed faecal culture, but not in pure culture. Among the 74 close contacts submitting faecal samples, 12 were aged 0-4 years, 13 aged 5-9 and 49 aged 10 years or older. *stx*_1 _was detected in mixed culture from two of these: one from a six year old child who was a sibling of a case and one from a mother of a case. s*tx *genes were not detected in samples from staff members.

*eae *without *stx *was detected in faecal cultures from 24 children in the day-care centre other than cases as well as from one sibling, from two parents and two staff members. Among these, 15 were detected in pure *E. coli *culture, and were thus classified as aEPEC, while *eae *without *stx *was detected only in mixed culture in 16 individuals.

Numbers of sampled individuals and results of PCR analysis of STEC and aEPEC genes (*stx*_1_, *stx*_2 _and *eae*) in culture of faecal samples from children, staff and close contacts are presented in Table 1.

**Table 1:** Results of PCR analysis of virulence genes (*stx*_1_, *stx*_2 _and *eae*) detected in samples from children, staff and close contacts of a day-care centre, Norway 2009

PCR results	Children attending day-care centre (n = 61)	Staff (n = 14)	Close contacts (n = 74)	Total (n = 149)
Pure culture				

STEC (*stx*_1_+*eae*)	16^a, b^	-	-	16
STEC (*stx*_1_, *stx*_2_+*eae*)	1	-	1	2
aEPEC (*eae*)	13^b^	2	-	15

Mixed culture only				

*stx*_1_+*eae*	1	-	2	3
*stx*_2_+*eae*	1	-	-	1
*eae *only	11	-	3	14

Total	43	2	6	51

^a ^Cases

^b ^For one case in whom both STEC and aEPEC was detected, only the STEC isolate is recorded in the table.

**Figure 1:**
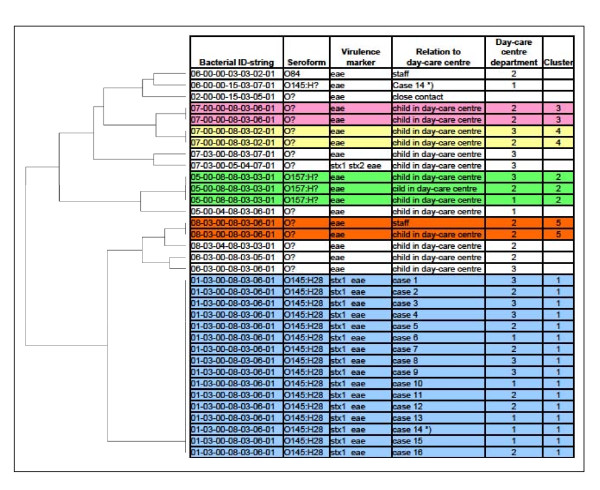
**MLVA profiles, serotypes and virulence markers among *E. coli *strains isolated from children and staff in day-care centre Norway, 2009**. Legend text: For each person only one isolate is listed, except for case 14 where both the outbreak STEC O145 strain and an aEPEC O145 strain with distinct different MLVA profiles (marked *) were detected. Each cluster is represented by a separate colour.

MLVA typing showed that isolates from all 16 cases had an identical profile: 01-03-00-08-03-06-01 (outbreak-profile). This profile has not previously been seen in *E. coli *isolated from humans, animals or foods in Norway. MLVA profiles from all *E. coli *strains isolated from 14 children in the day-care centre other than cases, from two staff members and from one close contact (total = 18), were distinctly different compared to the outbreak profile. A dendrogram presented in Figure 1 shows diversity in MLVA profiles and serotypes among all isolated *E. coli *strains.

Among the 35*E. coli *cultures isolated from 34 individuals, shown in the dendrogram, serotype O145 was detected exclusively from the case samples. However, from one single sample submitted by case 14, one *stx*-negative *E. coli *O145 strain with a distinct different MLVA profile was detected, in addition to the outbreak strain. Other serotypes detected were: O157: H? (three isolates) and O84 (one isolate), while 13 isolates did not belong to any common EPEC or STEC serotypes. Five clusters with mutual identical MLVA profiles were identified (cluster 1 - 5 shown in Figure 1). In addition to the STEC outbreak strain (cluster 1), four separate aEPEC clusters (cluster 2 - 5) were found. Two of the aEPEC clusters included children from more than one department; cluster 2 included four children from all three departments, and cluster 3 included children from two different departments. In contrast, both cluster 4 (including two children) and cluster 5 (including one child and one staff member) were represented only in department 2.

### Clinical and demographic results

Nine out of the 16 cases (56%) reported mild gastrointestinal symptoms, and none developed HUS or other serious symptoms. Demographic and clinical data related to cases are presented in Table 2.

**Table 2:** Demographic and clinical data related to cases in STEC O145 outbreak in child day-care centre Norway, 2009 (n = 16)

**Case number **^**a**^	Sex	Age (yrs)	Day-care centre department	Duration of shedding (days)	**Symptoms **^**b**^	**Level of diarrhoea **^**c**^	Date of onset (MM.DD)
1 (index)	m	1	3	57	b, f, m	5	09.28
2	f	3	2	71	n, m	2	10.11
3	m	3	3	14	n	4	09.28
4	m	2	1	23	n, f	1	10.07.
7	f	4	2	6	n	4	09.28
11	m	4	2	18	n	2	09.23 ^d^
12	f	2	2	22	n	2	10.07
13	f	1	1	0^e^	n, v	4	10.15
16	f	2	1	0^e^	n	2	09.23 ^d^
5	f	3	3	49	-	-	-
6	f	2	1	0^e^	-	-	-
8	m	3	3	22	-	-	-
9	m	3	3	47	-	-	-
10	m	1	1	64	-	-	-
14	m	2	1	0^e^	-	-	-
15	m	1	1	15	-	-	-

^a ^Cases were numbered chronologically in order of identification

^b ^Symptoms: n = non-bloody diarrhoea, b = bloody diarrhoea, v = vomiting, f = fever, m = malaise

^c ^1 = least severe, 5 = most severe

^d ^Date of onset reported by parents of case 12 and 16 as "during last two weeks of September". Median date of this period 09.23 is presented in this table, and in epidemic curve, Figure 2

^e ^Duration of shedding recorded as 0 if all follow up tests were negative.

Among symptomatic cases, one reported bloody diarrhoea (index case), eight reported non-bloody diarrhoea, one reported vomiting, two reported fever and three reported malaise. Level of diarrhoea was graded on a scale where 1 was least severe and 5 was most severe. One case reported grade 1, four cases reported grade 2, one case reported grade 3, three cases reported grade 4, and one case reported grade 5.

**Figure 2:**
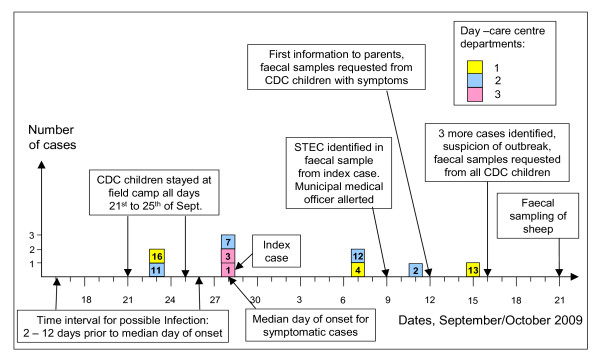
**Epidemic curve of STEC O145 outbreak in child day-care centre Norway, 2009 with dates of onset for symptomatic cases (n = 9)**. Numbers in case boxes refer to case number as listed in Table 2.

Among symptomatic cases, onset of disease occurred during the period of September 23^rd ^to October 15^th^. The median day of onset was September 28^th^. An epidemic curve illustrates onset of disease among symptomatic cases, and point of time for main events of the outbreak (Figure 2 and Table 3).

**Table 3:** Case attack rates within age groups

	Age (years)					Total all age groups
	1	2	3	4	5	
No. of children within age group	10	14	13	16	8	61
No. of cases within age group	4	5	5	2	0	16
Attack rate within age group	0.40	0.36	0.38	0.13	0	0.26

STEC O145 outbreak in child day-care centre Norway, 2009 (n = 16).

Overall attack rate among children in the day-care centre was 0.26. Attack rates within age groups are presented in Table 3. Fisher's exact test for the association between attack rate and aggregated age groups (1- 3 years and 4 - 5 years) showed that attack rate was significantly higher within the youngest age group (two-tailed p = 0.016). Attack rates and mean child age within departments are presented in Table 4.

**Table 4:** Attack rates and mean child age within day-care centre departments

	Department			Total all departments
	1	2	3	
No. of children within department	14	22	25	61
Mean child age within department	1.5	2.9	3.8	3.0
No. of cases within department	7	4	5	16
Attack rate within department	0.50	0.18	0.20	0.26

STEC O145 outbreak in child day-care centre Norway, 2009 (n = 16).

Duration of faecal shedding of *stx*_1_-positive STEC among cases is presented in Table 2. Median duration was 20 days. The two cases with longest duration, shed *stx*_1_-positive STEC for 63 and 71 days, respectively. From four cases *stx*_1_-positive STEC was detected in only one faecal sample. Children who tested positive were not allowed to attend the day-care centre until they had presented five consecutive faecal samples negative for *stx*_1_, according to national guidelines. The total number of days of faecal shedding for all cases was 408 days. Adding a minimum of 10 days per case for five follow up samples at intervals of two days, gives a minimum total of 568 days. Subtracting for weekends, the total number of weekdays which cases were not allowed to attend the day-care centre, can be estimated to minimum 406 days.

Among the 13 children in whom aEPEC was identified in faecal samples, two children belonged to the group of 16 children reporting a recent history of diarrhoea and first asked to submit faecal samples. The remaining 11 children and both staff members, in whom aEPEC was identified belonged to the group who did not report recent diarrhoea.

Among the group of 16 children reporting a recent history of diarrhoea that were first asked to submit faecal samples, neither STEC nor aEPEC was detected in faecal samples from nine children (56%). Samples were not tested for other pathogens.

### Environmental investigations of the day-care centre and exposure to risk factors

Environmental investigation revealed that food handling and general hygienic procedures in the day-care centre were acceptable and in accordance with legislative demands. Food items served to the children were industrially produced and commonly used items with a nationwide distribution. Child contact within departments was reported to be much more extensive compared to contact across departments. The day-care centre had established a field camp in a forest area near the city, and transported the children to this camp approximately once each week. The surrounding forest area was also used as pasture for a local sheep herd of 60 ewes and their lambs, and there was no fence to keep sheep out of the area where children stayed. The day-care centre children had normally no direct contact with sheep. However, sheep manure was regularly observed in the area, representing a source of potential faecal exposure from the sheep to the children during their normal activities at the field camp. The week September 21^st ^to 25^th ^was a special outdoor week, when all children in the day-care centre spent all attending days at the camp. In the period from September12^th ^to 25^th ^it rained every day, averaging 6.2 mm per day, and with maximum 23.9 mm on the 18^th^.

Compilation of data on exposures to children, collected from case interviews and from investigations in the day-care centre, did not indicate any common food-item or drink associated to STEC infection. Except for exposure to sheep manure during stay at the field camp, few of the cases had other contact to animals.

### Investigation among sheep

*E. coli *O145 was not detected in any of the faecal samples from sheep. During the first weeks of October the herd had been mustered, feeding regime was shifted from grazing over to dried grass, and a majority of lambs had been sent off for slaughtering.

## Discussion

In this study we describe an outbreak of *stx*_1_-positive STEC O145:H28 with mild symptoms among children in a day-care centre, with 16 cases identified by epidemiological investigations and use of molecular methods for microbial diagnosis and genotyping. Identification of identical MLVA profiles in *E. coli *isolates from all cases, previously not seen in any *E. coli *isolate from humans, animals or foods in Norway, strongly indicates the existence of a common outbreak source associated and restricted to this day-care centre. Sampling of all children and staff in the day-care centre, characterization of microbial diversity and clinical data from cases gave good insight into epidemiological aspects of this STEC O145 outbreak strain, and allowed for assessment of the epidemiological relationship between the STEC outbreak strain and aEPEC strains.

Only nine cases (56%) showed gastrointestinal symptoms, and none of the cases acquired HUS or other serious symptoms or adverse effects. Only the index case developed bloody diarrhoea. This pattern is consistent with observations from previous studies showing that infections with *stx*_1_-positive STEC are less frequently associated with bloody diarrhoea and HUS compared to infections with *stx*_2_-positive STEC. The observed overall attack rate (0.26) is within the range reported from similar outbreaks [33-36]. The attack rate varied between age groups and was significantly higher among children aged 1 - 3 years compared to children aged 4 - 5 years. Similar association of attack rate to age is reported by others [1], and is consistent with Norwegian surveillance for the period 2000 - 2009, which identified 124 domestically acquired STEC cases aged 1 - 3 years, compared to 14 cases aged 4 - 5 years. Higher attack rate in department 1 (0.50) compared to department 2 (0.18) and department 3 (0.20), is most likely attributable to lower child age in department 1 (mean 1.5 years) compared to department 2 (mean 2.9 years) and 3 (mean 3.8 years).

Observed median duration of shedding (20 days), is of similar magnitude to that reported by others [1,37]. Extremely prolonged shedding of 63 and 71 days, respectively, was observed in two cases. Incidents of STEC shedding from infected children for > 60 days have also been reported in other studies [36,38].

The results from the screening had considerable socioeconomic consequences, as many parents had to take compassionate leave to care for children in the period their child was not allowed to attend the day-care centre, in some instances for extensive periods of time. The total number of days of compassionate leave may be close to total number of days which cases were not admitted to the day-care centre, estimated to minimum 406 days. The low disease severity observed in this STEC outbreak underlines the need for reviewing routines and follow-up of STEC infections in Norway. STEC comprise a heterogeneous group of bacteria with considerable variation in virulence; and *stx*_1_-positive STEC, which are among the most frequent variants detected, has so far not been associated with HUS in Norway (unpublished data). A more graded response based on epidemiological and microbiological properties of the bacteria, and possibly taking into account the age of cases, may limit socioeconomic consequences described here and still ensure adequate handling of cases.

The clonal structure of isolated STEC and aEPEC strains, as illustrated by the dendrogram, shows that all aEPEC and *stx*_2_-positive STEC strains isolated from children, staff and close contacts had distinctly different MLVA profiles compared to case isolates, and showed genotypic and serologic diversity, indicating that children, staff and close contacts shedding these *E. coli *strains were not part of the O145:H28 outbreak. Further, these results confirmed that none of the aEPEC isolates represented the STEC outbreak strain that had lost *stx*-genes. Among four different aEPEC clones identified, two clones were represented by children or staff members from different departments. The observed clonal structure of isolated aEPEC cultures may reflect endemic aEPEC prevalence in the Norwegian child population.

Children and staff members, in whom aEPEC but not STEC was identified in faecal samples, were not interviewed. However, their symptomatic status may still be assumed based on whether samples were submitted from the group reporting a recent history of diarrhoea (including two aEPEC positive but STEC negative children), or from the remaining group not reporting a recent history of diarrhoea, and thus assumed to be asymptomatic (including 11 aEPEC positive but STEC negative children). Based on this assumption, only two of the 13 children (15.4%) and neither of the two staff members in whom aEPEC but not STEC was identified, showed gastrointestinal symptoms. The observed prevalence of aEPEC among children in this day-care centre is consistent with reported endemic prevalence of aEPEC in Norwegian children with mild diarrhoea [39], as well as in healthy children [25], and may therefore reflect a normal endemic level among children of this age. The observed low proportion of aEPEC-positive children showing gastrointestinal symptoms in our study is also consistent with findings in the studies referred to above. Any possible epidemiological association of aEPEC and *stx*_2_-positive STEC to the outbreak is uncertain. However, considering the possibility that this outbreak also included aEPEC strains transmitted from sheep or environment, can however not be excluded, and may be supported by the fact that two of the aEPEC clusters were found in children from different departments.

Although no analytical epidemiological study was conducted, several possible sources of infection and modes of transmission for the outbreak should be considered. Because foods served in the day-care centre were widely distributed brands, and based on the assumption that this outbreak was restricted to this specific day-care centre, food must be considered an improbable source of infection. Direct or indirect person to person transmission is a dominant mode of transmission for gastrointestinal infections within day-care centres [40], and should thus be considered. In this outbreak however, a low rate of secondary infections was observed. Among 74 close contacts who submitted faecal samples, of which 12 were children aged 0 - 4 years, and 13 aged 5 - 9 years,*stx*_1_-positive STEC was detected in only one child aged 6 years and in one adult, and not among 14 staff members. This indicates that the STEC outbreak strain had low potential for person to person transmission, even among children. Person to person transmission was therefore a less likely mode of infection for this outbreak.

From the group of 16 children reporting a recent history of diarrhoea and first asked to submit faecal samples, neither STEC nor aEPEC was detected in faecal samples from nine children. The causal agent for these symptomatic children is not clear. They may have been infected with STEC or aEPEC at an earlier stage, but ceased to shed at time of sampling. Or they may have been infected by other enteric pathogens not tested for in this investigation.

Due to a limited number of symptomatic cases, interpretation of the epidemic curve leaves no clear conclusion as to whether the source of infection was continuous or restricted to a time period. Hypothesizing the latter and considering September 28^th ^as median date of onset for symptomatic cases, and incubation period for STEC infection ranging from two to 12 days [1], the period of possible infection can be estimated to the interval ranging from September 16^th ^to 26^th ^as indicated in the epidemic curve, Figure 2.

Exposure to faecal pollution from sheep grazing around the field camp is one hypothesized mode of infection that fits with descriptive epidemiological data from the outbreak. The stay at the field camp each day during the week of September 21^st ^to 25^th^, where sheep grazed in the same area, and with steady rainfall during this and the preceding week, indicate peak exposure to children from sheep faecal microbes during this period, and falls within the estimated period of possible infection deduced from the epidemic curve. This hypothesis is supported by the absence of evidence for food or person to person transmission as probable mode of transmission for the outbreak. The hypothesis is further supported by other studies identifying direct or indirect contact to domestic ruminants as important risk factor for STEC infection of various serotypes, especially to small children [1,3,41,42], and due to this being a suspected source of infection in one other *E. coli *outbreak among Norwegian children during the year of 2009 [20].

Faecal sampling of sheep was performed subsequent to the recognition of the outbreak, approximately one month after the assumed peak exposure to children in the day-care centre. STEC colonization and shedding in ruminants may be intermittent, and is affected by shift of feeding [1,43,44]. such as it has occurred in this case. In STEC positive ruminant populations, younger individuals tend to have higher prevalence compared to adults [1,45]. Thus, delayed faecal sampling of the herd and subsequent to removal of lambs for slaughtering, reduced the probability of detecting STEC. As an alternative to rectal sampling, collection of manure from the ground could possibly have allowed for finding old faeces originating from lambs. This, however, would have been a far less sensitive sampling method because of dilution and lack of microbial survival, and rectal sampling was therefore applied. Considering these aspects, lack of STEC O145 detection from sheep does not give reason to rule out the hypothesis of sheep as source of infection. However, to our knowledge, human STEC O145 outbreaks caused by contact to sheep have not been previously reported, and in an unpublished prevalence study, STEC O145 was not detected in samples from 50 Norwegian sheep herds representing approximately 2500 sampled individuals. These observations, specifically concerning serotype O145, therefore do not support the hypothesis of contact to sheep as mode of infection for this outbreak.

Some aspects of the study design and results limit the ability to draw conclusions from this study. Severity of disease varied between cases and clinical data were based on subjective reports from parents, and thus vulnerable to misclassification. For this reason the case definition was based on microbiological and not clinical criteria. Uncertainties regarding clinical symptoms also affect the estimated onset of disease, which is the basis for the epidemic curve, which limits any conclusions drawn from this. All children in the day-care centre were potentially exposed to the main groups of presumed relevant risk factors such as food consumption, person to person contact, and animal contact, and data on exact exposure to these risk factors were not available. A cohort study comparing exposed and non-exposed children addressing these risk factors could therefore not be performed. Timing of faecal sampling from sheep was not optimal. Earlier sampling, and inclusion of a majority of lambs, would have increased the probability of detecting STEC from the sheep herd.

## Conclusions

We report an outbreak of *stx*_1_-positive STEC O145:H28 infection with mild symptoms among children in a day-care centre. The design of this cross-sectional study comprised extensive sampling of a well defined and easily accessible outbreak population, yielding a comparatively high number of identified cases, and allowing for extensive characterization of prevalence and genotypic and serologic diversity within STEC and aEPEC strains isolated from this population. Only nine of the 16 cases showed symptoms and only one case had bloody diarrhoea. No cases developed HUS or other serious complications to the infection. There was no conclusive evidence enabling identification of the source of infection, but there were indications of exposure to sheep faeces as one possible source of infection. Beyond the primary objectives of the outbreak investigation, this study has provided insight into microbial diversity and descriptive epidemiological aspects of a non-O157 STEC outbreak, which is less commonly reported, compared to O157 STEC outbreaks. The results from the screening of the day-care centre had great socioeconomic consequences, as all who tested positive had to stay home until cessation of shedding was proven, which for some cases meant extensive periods of time. The results indicate a need to evaluate the routines in Norway for follow-up of children with STEC infections. Since the pathogenic potential varies considerably among different STEC strains, a more graded response, based on both the epidemiological situation, the clinical picture of the patients and the microbiological properties of the bacteria, could possibly limit socioeconomic consequences and still ensure adequate handling of cases.

## Competing interests

The authors declare that they have no competing interests.

## Authors' contributions

EW has been responsible for the outbreak investigation, and has drafted the manuscript. JEA was responsible for microbial investigation of human faecal samples and contributed to epidemiological assessments. LV contributed to epidemiological and public health assessments. BAL was responsible for multilocus variable number of tandem repeat analysis. TB was responsible for all microbial investigations of faecal samples from sheep as well as AIMS analysis of cultures isolated from human faeces, and contributed to veterinary epidemiological assessments. All authors have read and approved the final manuscript.

## Pre-publication history

The pre-publication history for this paper can be accessed here:

http://www.biomedcentral.com/1471-2334/11/238/prepub

## Supplementary Material

Additional file 1 **Supplementary table**. Primers employed in PCR analysis of human faecal samples. STEC O145 outbreak in child day-care centre Norway, 2009 Click here for file
