# Supplementary material for: Investigation of an Escherichia coli O145 outbreak in a child day-care centre - extensive sampling and characterization of eae- and stx1-positive E. coli yields epidemiological and socioeconomic insight
Source: BMC Infect Dis. 2011 Sep 8;11:238. doi: 10.1186/1471-2334-11-238 (PMC3188501; doi:10.1186/1471-2334-11-238)
Supplement: Additional file 1 — Supplementary table. Primers employed in PCR analysis of human faecal samples. STEC O145 outbreak in child day-care centre Norway, 2009 [file 1471-2334-11-238-S1.DOC]

**Supplementary table**

| Primer/ probe | Oligonucleotide sequence (5’-3’) |
| --- | --- |
| eaeF | TTC ATT GAT CAG GAT TTT TCT GG |
| eaeR | GCT CAT GCG GAA ATA GCC |
| eae probe | FAM-ATA GTC TCG CCA GTA TTC SCC MCC AAT ACC-LC640 |
| stx1F | AAA TCG CCA TTC GTT GAC TAC TTC T |
| stx1R | CCA TTC TGG CAA CTC GCG ATG CA |
| stx1 probe | FAM-AAC CTC ACT GAC GCA GTC TGT GGC AAG AGC-LC640 |
| stx2F | CAG TCG TCA CTC ACT GGT TTC ATC |
| stx2R | GGA TAT TCT CCC CAC TCT GAC AC |
| stx2 probe | HEX-CTG TCA CGG CAG AAG CCT TAC GCT TCA GGC-LC640 |
